# Differentially Expressed miRNAs in Ulcerative Colitis and Crohn’s Disease

**DOI:** 10.3389/fimmu.2022.865777

**Published:** 2022-06-06

**Authors:** Reza Yarani, Ali Shojaeian, Oana Palasca, Nadezhda T. Doncheva, Lars Juhl Jensen, Jan Gorodkin, Flemming Pociot

**Affiliations:** ^1^Translational Type 1 Diabetes Research, Department of Clinical Research, Steno Diabetes Center Copenhagen, Gentofte, Denmark; ^2^Interventional Regenerative Medicine and Imaging Laboratory, Department of Radiology, Stanford University School of Medicine, Palo Alto, CA, United States; ^3^Research Center for Molecular Medicine, Hamadan University of Medical Sciences, Hamadan, Iran; ^4^Novo Nordisk Foundation Center for Protein Research, University of Copenhagen, Copenhagen, Denmark; ^5^Center for Non-Coding RNA in Technology and Health, University of Copenhagen, Copenhagen, Denmark; ^6^Department of Veterinary and Animal Sciences, University of Copenhagen, Copenhagen, Denmark; ^7^Copenhagen Diabetes Research Center, Department of Pediatrics, Herlev University Hospital, Herlev, Denmark; ^8^Department of Clinical Medicine, Faculty of Health and Medical Sciences, University of Copenhagen, Copenhagen, Denmark

**Keywords:** miRNA, ulcerative colitis, Crohn’s disease, inflammatory bowel disase, Transcriptomics

## Abstract

Differential microRNA (miRNA or miR) regulation is linked to the development and progress of many diseases, including inflammatory bowel disease (IBD). It is well-established that miRNAs are involved in the differentiation, maturation, and functional control of immune cells. miRNAs modulate inflammatory cascades and affect the extracellular matrix, tight junctions, cellular hemostasis, and microbiota. This review summarizes current knowledge of differentially expressed miRNAs in mucosal tissues and peripheral blood of patients with ulcerative colitis and Crohn’s disease. We combined comprehensive literature curation with computational meta-analysis of publicly available high-throughput datasets to obtain a consensus set of miRNAs consistently differentially expressed in mucosal tissues. We further describe the role of the most relevant differentially expressed miRNAs in IBD, extract their potential targets involved in IBD, and highlight their diagnostic and therapeutic potential for future investigations.

## Introduction

Inflammatory bowel disease (IBD) is an idiopathic, chronic inflammation that primarily affects the gastrointestinal tract. IBD patients experience frequent hospital admissions, many operations, and poor quality of life due to the disease complications (1, 2). Like many other immune-related diseases, the etiology of IBD is not well understood. However, it is generally believed to be a multifactorial disease where environmental factors, genetics, immune dysregulation, and microbiome dysbiosis trigger an inappropriate immune response in lamina-propria, which challenges mucosal homeostasis (3).

Ulcerative colitis (UC) and Crohn’s disease (CD) are the two major types of IBD. While CD shows a patchy transmural inflammatory pattern, UC is mainly limited to the innermost layers and rarely affects other layers of the intestine wall (1, 2). CD is associated with many pathophysiological complications, and its clinical symptoms vary according to the disease location (4). UC is more prevalent and mainly affects the colon (rectum) and generally has a milder course, with patients less prone to disease complications (5, 6). Genome-wide association studies (GWAS) identified 245 unique IBD loci. These susceptible loci are crucial in defining the disrupted intestinal immune system and disease pathways and constitute a solid genetic component of IBD (7–9).

Advances in IBD genetics, high-throughput sequencing technologies, and transcriptome studies provide new insights associated with noncoding RNAs, including long noncoding RNAs (lncRNAs) (10) and microRNAs (miRs or miRNAs) in various diseases (11, 12). Differentially expressed miRNAs are highly correlated with inflammatory and autoimmune disorders, including psoriasis (13), rheumatoid arthritis (14), multiple sclerosis (15), and IBD (16, 17). Mature miRNAs are short (~22 nt long) single-stranded noncoding RNAs derived from pre-miRNA hairpins (typically ~80 nt), and many of these are further processed from primary miRNA transcripts (pri-miRNA) of several hundred nucleotides when multiple pre-miRNAs are contained. The pri-miRNA can be intergenic or of intronic origin nucleotides and can be evolutionarily conserved. MiRNAs are involved in regulating gene expression post-transcriptionally (17–19), where the mature miRNA binds to its target typically with a seed sequence of 6 nucleotides from position 2-7 and with the remaining part binding often with a few nucleotide bulges.

Various studies indicate that differentially expressed miRNAs affect mRNA at several levels of regulation: transcriptional, post-transcriptional, chromatin modification, and genomic imprinting. miRNAs can affect biological processes through endogenous RNA competition, regulation of RNA transcription, protein sponges, and translation regulation. These regulations can cause decreased stability and translational repression that affects various biological functions, including proliferation, migration, cell signaling, autophagy, and apoptosis (3, 17, 20, 21). It is estimated that miRNAs regulate more than 60% of the mRNA through complementary pairing at 3′ untranslated regions (UTRs) (20). miRNAs are not only acting as local regulators within the cells; they also can be found in places far from their origin and are directly or indirectly involved in virtually all types of regulation of biological processes in living organisms (17, 21).

Furthermore, some miRNAs are stable in body fluids such as serum, plasma, urine, saliva, and other tissues (22–25). Many efforts are currently ongoing to identify differentially expressed miRNAs in IBD as biomarkers. Since the expression of differentially expressed miRNA in IBD and many other diseases seems to happen early in the disease, the evaluation of circulating miRNA or tissue-specific levels could be helpful for early diagnosis and successful treatments. Thus, it is highly important to study miRNA-expression profiles and their target genes as biomarkers for diagnosis, prognosis, progression, and treatment response.

This review presents an overview of current knowledge on differentially expressed miRNAs in IBD patients’ mucosal tissues and peripheral blood. To enrich the findings from the literature, we combined the literature curation with a meta-analysis of publicly available miRNA high-throughput datasets in mucosal tissues. We further discuss the importance of the most relevant miRNAs in the disease based on the available knowledge and suggest the miRNA participation role in developing chronic inflammation that characterizes pathogenesis. Finally, we discuss the relevance of miRNA differential expression for prediction/early diagnosis, disease progression and treatment responses, and the obstacles in the way.

### Differentially Expressed miRNAs in IBD Patient’s Mucosal Tissues

#### Ulcerative Colitis

The first miRNA profiling study in IBD was performed in 2008 and compared biopsy samples from patients with active UC (aUC), inactive UC (iUC), chronic active CD (aCD), microscopic colitis, infectious colitis, and irritable bowel syndrome with healthy controls (26). Eleven miRNAs were differentially expressed in patients with aUC compared to the controls. miR-192-5p, miR-375-3p, and miR-422b-5p were significantly downregulated, and miR-16-5p, miR-21-5p, miR-23a-5p, miR-24-3p, miR-29a-3p, miR-126-3p, miR-195-5p, and let-7f-5p were significantly upregulated (26).

Following this pioneering observation, subsequent studies have identified many new miRNAs while reconfirming already identified ones. It is not surprising that the findings are not consistent as many variables differ between studies, including treatment, inflammatory status, disease duration, anatomical biopsy locations, different healthy control cohorts, and miRNAs profiling platforms. Regardless of these differences, several miRNAs are frequently reported as being differentially expressed. miR-21-5p (26–32), miR-155-5p (27, 29, 33–35), miR-31-5p (27, 31, 33, 36), miR-146a-5p (27, 30, 32, 34), miR-126-3p (26, 28, 32), miR-29a-3p (26, 36), miR-16-5p (26, 28), miR-223-3p (32, 35) and miR-24-3p (26, 30) showed to be constantly upregulated while miR-192-5p (26, 28, 30), miR-141-3p (32, 37), and miR-375-3p (26, 30) were downregulated in UC biopsies when compared to control biopsies (in at least two independent studies). Also, many miRNAs showed differential regulation when inactive UC is compared with active UC and control (**Table 1**).

**Table 1:** Differentially expressed miRNAs in human UC colonic tissue based on literature review.

Up-regulated	Down-regulated	Other finding/Comments	Method	Source	Group/N	Country	Ref
miR-16-5p, -21-5p, -23a-5p, -24-3p, -29a-3p, -126-3p, -195-5p, let-7f-5p	miR-192-5p, -375-3p, and -422b-5p	Macrophage inflammatory peptide (MIP)-2 alpha which is a chemokine expressed by epithelial cells showed to be the target of miR-192-5p.	Microarray qRT-PCR	Sigmoid and colon	aUC/15 iUC/15 CO/15	USA	(26)
miR-21-5p, -203-5p, -126-3p and 16-5p	miR-320-5p, -192-5p	miR-16, -143, and -145 are expressed in response to DNA damage	qRT-PCR	Colon	UC/5 CO/5	USA	(28)
Non-inflamed: miR-15a-5p, -26a-5p, -29a-3p, -29b-5p, -30c-5p, -126-3p, -127-3p, -324-3p Inflamed: miR-7-5p, -26a-5p, -29a-5p, -29b-5p, -31-5p, -126-3p, -127-3p, 135b-5p, 324-3p	miR-199b-5p, -370-5p in both aUC and iUC	Commonly dysregulated in UC and CD: miR-26a-5p,-29a-3p,-29b-5p,-30c-5p,-126-3p,-127-3p,-196a-5p,-324-3p	qRT-PCR	Colon	iUC/8 CO/10	France	(36)
miR-21-5p, -155-5p	–	Other up-regulated miRNAs are (not significant): let-7a-5p,l et-7c-5p, let-7d-5p, let-7g-5p, miR-923-5p	Microarray qRT-PCR	Colon	Microarray: UC/2 qRT-PCR: UC/12 CO/12	Japan	(29)
aUC vs iUC: miR-650-5p, -548a-3p	aUC vs iUC: miR-630-5p, -489-5p, -196b-5p	–	Microarray qRT-PCR	Sigmoid and colon	aUC/9 iUC/9 CO/ 33	Italy	(38)
aUC vs CO: miR-21-5p, -31-5p, -146a-5p, -155-5p, -650-5p aUC and iUC vs CO: miR-675-5p	aUC vs CO: miR-196b-5p, -196b-3p, -200c-3p aUC and iUC vs CO: miR-378a-5p, -196b-5p, -10b-5p	miR-200c-3p directly regulates IL8 and CDH11 expression (regulators of immune and barrier integrity) and can be used for therapeutic purposes.	Affymetrix qRT-PCR	Colon	UC/17 CO /10	Belgium	(27)
miR-24-3p, -142-3p, -146a-5p, -21-5p, let-7i	miR-192-5p, -194-5p, -200b-5p, -375-3p	Rectal miR-24-3p was increased 1.47-fold in UC compared to CD samples.	qRT-PCR	Rectum	UC/18 CO/20	Netherlands	(30)
miR-19a-3p, -21-5p, -31-5p, -101-5p	–	miR-21-5p, -31-5p, and -142-3p were significantly upregulated and miR-142-5p was significantly downregulated in saliva of UC patients.	Microarray qRT-PCR	Colon	UC /41 CO/35	USA	(31)
miR-155-5p, -146a-5p	miR-122-5p	–	qRT-PCR	Colon	UC/10 CO/23	Hungary	(34)
miR-18a-5p, -21-5p, -31-5p, -99a-5p, -99b-5p, -125a-5p, -126-3p, -142-5p, -146a-5p, -223-3p	miR-141-3p, -204-5p	Upregulation of miR-31-5p, -125a-5p, -146a-5p and -223-3p, and downregulation of miR-142-3p in the inflamed mucosa of pediatric UC compared to children with CD was observed	qRT-PCR	colon biopsies	UC/32 CO/11	Hungary	(32)
–	miR-141-3p	miR-141-3p is important in inflammation by inducing CXCL5 upregulation in UC patients	qRT-PCR Western Blot	sigmoid and colon biopsies	aUC /15 CO/ 13	china	(37)
miR-125b-5p, -155-5p, -223-3p, -138-5p	miR-378d-5p	miR-200a-5p did not change significantly in the inflamed samples when compared with non-inflamed and controls.	Microarray qRT-PCR	colon biopsies	UC/8 CO/8	India	(35)
miR-31-5p, -155-5p	–	IL13Rα1 is downregulated in the inflamed UC mucosa and both miRNAs are targeting its 3UTR	qRT-PCR Western Blot	sigmoid and colon biopsies	aUC/11 CO/11	UK	(33)

All miRNAs are from comparison between the disease and healthy individual, unless otherwise stated.

aUC, active UC; iUC, inactive UC; CO, Control; N, Numbers per Group.

#### Crohn’s Disease

In another pioneering study in 2010, Fasseu et al. identified 14 and 23 miRNAs differentially expressed (0.001< p <0.05) in iUC and inactive CD (iCD) patients, respectively (**Tables 1**, **2**). Among them, 8 were commonly differentially expressed in iUC and iCD (miR-26a-5p, miR-29a-3p, miR-29b-5p, miR-30c-5p, miR-126-3p, miR-127-3p, miR-196a-5p, miR-324-3p). Further analysis showed that miR-26a-5p, miR-29b-5p, miR-126-3p, miR-127-3p, and miR-324-3p had coordinated differential regulation in the non-inflamed and inflamed colonic mucosa of IBD patients. On the other hand, miR-196b-5p, miR-199a-3p, miR-199b-5p, miR-320a-5p, miR-150-5p, and miR-223-3p demonstrated significant difference when non-inflamed UC and CD colonic biopsies were compared. Based on this screening, the authors suggested an important role of miRNAs in the inflammation at onset and/or relapse of IBD patients with quiescent mucosal tissues (36).

**Table 2:** Differentially expressed miRNAs in human CD colonic tissue based on literature review.

Up-regulated	Down-regulated	Other finding/Comments	Method	Source	Group/N	Country	Ref
Non-inflamed miR-7-5p, -26a-5p, -30b-5p, -30c-5p, -155-5p, -127-3p, -223-3p, -324-3p Inflamed: miR-26a-5p, -29b-5p, -126-3p, -155-5p, -127-3p, -185-5p, -196a-5p, -324-3p, -378-5p	miR-130b-5p in inflamed CD	Commonly dysregulated in UC and CD: miR-26a-5p, -29a-3p, -29b-5p, -30c-5p, -126-3p, -127-3p, -196a-5p,-324-3p	qRT-PCR	Colon	iCD/8 CO/10	France	(36)
colonic CD vs CO: miR-23b-3p, -106a-5p, and -191-5p active ileal CD vs CO: miR-16-5p, -21-5p, -223-3p and -594-5p	colonic CD vs CO: miR-19b-3p, -629-5p	Ten intestine region-specific miRNAs were identified. miR-22-5p, -31-5p, and -215-5p were significantly increased in the terminal ileum as compared to all four colonic regions	Microarray qRT-PCR	Terminal ileum, cecum, transverse colon, sigmoid, and rectum	Sigmoid CD/5 Terminal ileum CD/6 CO/13	USA	(39)
aCD versus iCD: miR-18a-3p, -629-3p, , let-7b, -140-3p	aCD versus iCD: miR-422a-5p, -885-5p, -328-5p		Microarray qRT-PCR	Colon	aCD/9 iCD/9	Italy	(38)
miR-19b-3p, -23b-3p, -106a-5p, -629-5p	–	CD vs UC: Significant differential expression of miR-19b-3p, -106a-5p, -629-5p Average expression of these three miRNAs and miR-23b-3p and -191-5p was significantly different between intermediate colitis and CD No significant difference was detected between UC, intermediate colitis and controls	qRT-PCR	Colon	CD/14 UC/12 Intermediate/16 CO/11	USA	(40)
miR-142-3p, -146a-5p, -21-5p, let-7i	miR-194-5p, -200b-5p, -192-5p and -375-3p	Rectal miR-24-3p correctly classified 84.2% of patients, with a sensitivity of 83.3% and specificity of 85.7%.	qRT-PCR	Colon	CD/12 CO/20	USA	(30)
Non-inflamed vs CO: miR-495-5p Inflamed vs non-inflamed: miR-361-3p	Inflamed vs CO: miR-192-5p Non-inflamed vs CO: let-7b-5p Inflamed vs non-inflamed: miR-124-3p		Microarray qRT-PCR	Terminal ileum	CD/16 CO/10	China	(41)
In B2 and/or B3: miR-31-5p, -215-5p, -223-3p	In B1: miR-150-5p In B2 and/or B3: miR-149-5p, -196b-5p, -203-5p	B1: nonstricturing and nonpenetrating (n=8) B2: structuring (n=6) B3: penetrating/fistulizing (n=7) The expression level of miR-31-5p was the most significant in both B2 and B3	RNA-Seq qRT-PCR	Colon	Sequencing: CD/21 CO/14 Validation: CD/20 CO/15	USA	(42)
miR-31-5p, -101-5p and -146a-5p	miR-375-3p	miR-101 in CD patients’ saliva was significantly upregulated. ATG16L1 as a regulatory target of miR-142-3p and miR-93-5p	Microarray qRT-PCR	Colon	CD /42 CO/35	USA	(31)
miR-146a-5p and -155-5p Inflamed CD vs CO: miR-122-5p (not significant)	Inflamed CD vs intact CD: miR-122-5p	miR-146a and -155 have also been connected to TLR pattern recognition receptor family. TNF-α treatment in HT-29 cells increased the expression of miR-146a-5p and -155-5p, but not miR-122-5p.	RNA-Seq qRT-PCR	Colon	Intact pCD/14 Inflamed pCD/24 CO/23	Hungary	(34)
inflamed vs intact duodenal mucosa: miR-146a-5p inflamed CD vs CO duodenal mucosa: miR -155-5p		TGF-β treatment had no effect on miR-146a-5p miR-122-5p expression in duodenal epithelial cells, while significant downregulation was detected for miR-155-5p.	qRT-PCR	Duodenal	intact CD/10 inflamed CD/10 CO/10	Hungary	(43)
Inflamed vs CO: miR-18a-5p, -21-5p, -31-5p, -99a-5p, -99b-5p, -100-5p, -125a-5p, -126-3p, -142-5p, -142-3p, -146a-5p, -150-5p, -185-5p, and -223-3p Non-inflamed vs CO: miR-18a-5p, -20a-5p, -21-5p, -31-5p, -99a-5p, -99b-5p, -100-5p, -125a-5p, -126-3p, -142-5p, -146a-5p, -185-5p, -204-5p, -221-5p, and -223-3p	Inflamed vs CO: miR-20a, -141-3p, -204-5p Inflamed vs non-inflamed and CO: miR-141-3p, miR-204-5p	miR-31-5p, -125a-5p, -142-3p-5p, and -146a-5p showed alter expression between the inflamed mucosa of CD and UC	qRT-PCR	Colon	RNA-Seq: CD/4 CO/4 Validation: CD/15 CO/11	Hungary	(34)
miR-193b-3p, -19a-3p, let-7I, let-7I-3p, -1273D-5p, -886-5P, -668-5p, -720-5p, -455-3P, -3138-5p, -612-5p, -551B-5p, -4264-5p, -24-3p	miR-3194-5p, -196A-5p, -192-5p, -200A-5p, -192-3p, -1913-5p, -378b-5p, -323b-3P, -3150-5p, -422A-5p, -611-5p, -3184-5p, -4284-5p, -129-3p	miR-4284-5p, -3194-5p and -21-5p interact with JAK-STAT signaling and innate immune system	Microarray qRT-PCR	Colon	CD/15 CO/15	Italy	(44)
miR-144-5p, -451-5p, -31-5p and -142-3p iCD vs CO: miRplus-F1195 and -150-5p	miR-1973-5p, -1205-5p, -5481-5p, -491-5p -3p CD and iCD vs CO: miR-1205-5p downregulation	Inhibition of C10orf54 expression by miR-16-1-5p is one of the main causes of CD	Microarray qRT-PCR	Ascending colon	CD/7 iCD/7 CO/7	USA	(45)
miR-31-5p		a dramatic and highly significant upregulation (~60-fold) of miR-31-5p in IL patients compared with control	RNA-Seq qRT-PCR	Ascending colon	CD/76 CO/51	USA	(46)
miR-21-5p, -223-5p, -1246-5p	miR-30c-5p, -378-3p		Microarray qRT-PCR	Ileal colon	CD/18 CO/12	Belgium	(47)
miR-223-3p	miR-194-5p, -10b-5p, -215-5p, -192-5p, -10a-5p, -582-5p	miR-31-5p expression was location driven suggest a CD location subtypes	NanoString	Ileal Colon	CD/23 CO/ 38	Canada	(48)

All miRNAs are from comparison between the disease and healthy individual, unless otherwise stated.

aCD, active CD; iCD, inactive CD; CO, Control; N, Numbers per Group.

Succeeding studies have identified several miRNAs consistently shown to be differentially expressed between CD and control biopsies, including always upregulated miR-146a-5p (30–32, 34, 43), miR-21-5p (30–32, 39, 47), miR-31-5p (31, 32, 42, 45, 46), miR-223-3p (32, 39, 42, 48), miR-142-3p (30, 32, 45), let-7i-5p (30, 44), miR-23b-3p (39, 40), miR-106a-5p (39, 40) and constantly downregulated miR-192-5p (41, 44, 48), miR-194-5p (30, 48) and miR-375-3p (30, 31). There are also miRNAs with conflicting results including miR-150-5p (up in (32, 45), down in (42)), miR-19b-3p (up in (40), down in (39)), miR-215-5p (up in (42), down in (48)), and miR-629-5p (up in (40), down in (39)). Moreover, several miRNAs showed differential regulation when iCD compared with aCD and control (**Table 2**).

### Differentially Expressed miRNAs in IBD Patient’s Peripheral Blood

#### Ulcerative Colitis

Similar to the findings in tissue biopsies, miRNAs are also differentially expressed in the peripheral blood of UC patients. In a first study, Wu et al. compared the circulating miRNA profile of whole blood of aUC and iUC patients and healthy individuals (49). Their microarray investigation showed a significant increase in the expression level of twelve miRNAs, while one, miR-505-3p, showed a significant decrease when comparing patients with aUC with healthy controls. miR-505-3p expression was decreased around 7-fold in active outpatient blood. In contrast, 3.1- and 5.2-fold expression increases were demonstrated in the blood of the active UC patients for miR-103-2-3p and miR-362-3p, respectively. Furthermore, a comparison between the circulating miRNA in the peripheral blood of UC patients with healthy individuals revealed a significant increase in the expression level of the miR-28-5p, miR-151a-5p, miR-199a-5p, miR-340-3p, and miRplus-E1271 in patients with aUC but not in iUC. Wu et al. further demonstrated that miRs-103-2-3p, miR-362-3p, and miR-532-3p are upregulated in both aUC and iUC. Following this initial study, in attempts to identify circulating miRNAs that contribute to UC development and to find proper biomarker candidates, many studies have been performed. From these studies miR-223-3p (3, 31, 38, 50, 51), miR-142-5p (31, 38, 52), miR-16-5p (50, 53, 54), miR-151a-5p (49, 54), miR-199a-5p (49, 54), miR-19a-3p (31, 38), miR-24-3p (38, 52), miR-28-5p (49, 54), miR-30e-5p (38, 51), miR-362-3p (49, 55) showed consistent upregulation in at least two independent studies, whereas none of the downregulated miRNAs had been validated in more than one study (possibly due to biases in which miRNAs are picked for validation). Moreover, miR-21-5p (up in (49, 50), down in (31)), miR-146a-5p (up in (56), down in (31)), miR-150-5p (up in (56), down in (38)), miR-188-5p (up in (57), down in (51)), miR-199a-3p (Up in (38), down in (56)) showed inconsistent differential regulation between different studies. miRNAs differential regulation was also detected when iUC was compared with aUC and control. miR-362-3p is the only miRNA that shows upregulation in two independent studies when iUC was compared with healthy control (49, 55) (**Table 3**).

**Table 3:** Differentially expressed miRNAs in human UC peripheral blood based on literature review.

Up-regulated	Down-regulated	Other finding/Comments	Method	Source	Group/N	Country	Ref
miR-28-5p, -151a-5p, -199a-5p, -340-3p, and miRplus-E1271 aUC and iUC: miR-103-2-3p, -362-3p, and -532-3p	aUC and iUC: miR-505-3p UC vs CD: miR-505-3p	UC specific: miRplus-E1153 miRs-28-5p, -103-2-3p, -149-3p, -151a-5p, -340-3p, -505-3p, -532-3p, and miR-plus-E1153, were able to distinguish aCD from aUC	Microarray RT-qPCR	Peripheral blood	aUC/13 iUC/10 CO/13	USA	(49)
miR-188-5p, -422a-5p, -378-5p, -500-5p, -501-5p, -769-5p, -874-5p		Classifier measurements demonstrated a predictive score of 92.8% accuracy, 96.2% specificity and 89.5% sensitivity in stratifying UC patients from controls using these miRNAs panel.	Microarray RT-qPCR	Peripheral blood	UC/20 CO/20	USA	(57)
miR-16-5p, -21-5p, -28-5p, -151a-5p, -155-5p and -199a-5p			RT-qPCR	Peripheral blood	UC/88 CO/162	Greece	(54)
miR-760-5p, -423-5p, -128-5p, -196b-5p, -103-5p, -221-5p, -532-5p, -15b-5p, -27a-5p, let-7g-5p, -93-5p, let-7d-5p, -598-5p, -142-5p, let-7e, -223-3p, -374b-5p, -19a-3p, -345-5p, -199a-3p, -24-3p, -30e-5p, -29a-3p, -28-3p aUC vs iUC: miR-650-5p and -548a-3p	miR-150-5p aUC vs iUC: miR-630-5p, -489-5p, and -196b-5p	miR-127-3p, -491-5p, -18a-5p, -145-5p, let-7b-5p, -185-5p, -29c-5p, -19b-3p, -20b-5p, -106a-5p, -17-5p, -222-5p, -135a-3p were common between CD and UC	TaqMan human miRNA array RT-qPCR	Serum	aUC/9 iUC/9 CO/33	Italy	(38)
miR-16-5p, -34b-3p UC vs CD: miR-377-3p, -1247-5p miR-34b-3p, -484-5p, -574-5p in both CD and UC	miR-99b-5p	miR16-5p regulates HMGA1/2 and ACVR2a while miR-34b regulates HNF4A, NOTCH1, c-MET/HGFR and CAV1 and miR-99b-5p regulates RAVER2 and mTOR which are all IBD-risk genes	Microarray	Peripheral blood	UC/36 CO/38	Germany	(53)
miR-595-5p, -1246-5p, -142-5p, -143-5p, -24-3p aUC vs iUC: miR-1246-5p and miR-595-5p		NCAM-1 and FGFR2 are two potential targets of miR-595 miR-1246 indirectly activates the proinflammatory nuclear factor of activated T cells	Microarray RT-qPCR	Serum	UC/62 CO/58	New Zealand	(52)
miR-223-3p, -23a-3p, -302-3p, -191-5p, -22-3p, -17-5p, -30e-5p, -148b-3p, -320e-5p	miR-1827-5p, -612-5p, -188-5p UC vs iUC: miR-4454-5p, -223-3p, -23a-3p, -148b-3p, -320e-5p, -4516-5p	Positive disease severity correlation of miR-223-3p, -4454, -23a-3p, -148b-3p, -320e-5p, and -4516-5p miR-4454-5p, -223-3p, -23a-3p, and-320e-5p showed higher sensitivity and specificity values (70% and 68%, 79% and 72%, 79% and 68%, and 67% and 67%, respectively) than C-reactive protein (37% and 95%)	Nanostring Analysis	Peripheral blood and serum	UC/24 iUC/22 CO/21	USA	(51)
miR-19a-3p, -101, -142-5p, -223-3p, -375-3p, and -494-5p	miR-21-5p, -31-5p, and -146a-5p	miR-21-5p, -31-5p, and miR-142-3p were significantly upregulated and miR-142-5p was significantly downregulated in saliva of UC patients.	Microarray qRT-PCR	Peripheral blood	UC /41 CO/35	USA	(31)
miR-223-3p		miR-223-3p demonstrated high Spearman r value in detecting the disease activity	RT-qPCR FC: 2.8	Serum	UC/50 CO/50	China	(3)
miR-29b-3p, -122-5p, -150-5p, -192-5p, -194-5p, -146a-5p, -375-3p	miR-199a-3p, -148a-3p	miRNA used in this study were discovered in IL10−/− mice model of UC and tested for orthologues in human. UC stratified from CO with 83.3% prediction rate	miRCURY LNA RT-qPCR Prediction	Serum	UC/12 CO/12	USA	(56)
aUC vs CO: miR-106a-5p iUC vs CO: miR-106a-5p and -362-3p		The expression level of miR-362-3p showed to be higher in UC vs CO but not significant.	RT-qPCR	Peripheral blood	aUC/20 iUC/12 CO/32	Iran	(55)
miR-16-5p, -21-5p and -223-3p	–	miR-155 expressed higher in CD than UC In remission group expression of miRNAs was dependent on disease activity	RT-qPCR	Serum	UC/15 CO/20 Remission UC/8	Germany	(50)

All miRNAs are from comparison between the disease and healthy individual, unless otherwise stated.

aUC, active UC; iUC, inactive UC; aCD, active CD; iCD, inactive CD; CO, Control; N, Numbers per Group.

#### Crohn’s Disease

One of the first studies using whole blood for distinguishing CD patients from normal healthy individuals using miRNA profile was done by Wu et al. (49). Comparing the circulating miRNA of the aCD patients with healthy controls showed a significant increase in the expression of five miRNAs and a significant decrease in two others. Among them, miR-362-3p showed the most significant difference in expression of a 4.7-fold increase. Interestingly the expression of miR-340-3p showed a significant increase, and miR-149-3p showed a significant decrease in both active and inactive CD patients compared to the healthy controls.

Subsequent studies found miR-16-5p (38, 50, 54, 58), miR-484-5p (53, 58, 59), miR-362-3p (49, 54, 55), miR-106a-5p (54, 55, 58), miR-532-3p (49, 54), miR-30e-5p (58, 60), miR-223-3p (3, 50), miR-21-5p (50, 58), miR-200c-3p (54, 61), miR-199a-5p (49, 54), miR-195-5p (38, 58), miR-142-5p (52, 53), miR-140-5p (38, 58) to be consistently upregulated in CD patients in comparison with healthy controls (in at least two independent studies). However, similar to the UC studies, based on the lists manually curated from literature, no circulating miRNA is always downregulated when CD is compared to healthy controls (in more than one study). This could be because the main focus for blood-based biomarker discovery is on the upregulated miRNAs, not the downregulated ones. There are also miR-574-5p (up in (53), down in (60)) and miR-192-5p (up in (58), down in (60)) that were shown to be differentially expressed inconsistently between studies. Moreover, several circulating miRNAs showed differential regulation when iCD compared with aCD and control (**Table 4**).

**Table 4:** Differentially expressed miRNAs in human CD peripheral blood based on literature review.

Up-regulated	Down-regulated	Other finding/Comments	Method	Source	Group/N	Country	Ref
miR-199a-5p, -362-3p, -532-3p, miRplus-E1271 aCD and iCD: miR-340-3p	miRplus-F1065 aCD and iCD: miR-149-3p	miR-199a-5p, -362-3p, -340-3p, -532-3p and miRplus-E1271 common in both CD and UC	Microarray RT-qPCR	Peripheral blood	aCD/14 iCD/5 CO/13	USA	(49)
miR-16-5p, -195-5p, -106a-5p, -20a-5p, -30e-5p, -140-5p, -484-5p, -93-5p, -192-5p, -21-5p and let-7b-5p		Area under the ROC curve values of 0.82 to 0.92 sensitivities of 70% to 83% and specificities of 75% to 100%	TaqMan Human MicroRNA Arrays RT-qPCR	Serum	CD/46 CO/32	USA	(58)
miR-16-5p, -23a-5p, -29a-3p, -106a-5p, -107-5p, -126-3p, -191-5p, -199a-5p, -200c-3p, -362-3p and -532-3p			RT-qPCR	Peripheral blood	CD/128 CO/162	Greece	(54)
miR-27a-5p, -140-3p, -140-5p, -16-5p, -195-5p aCD vs iCD: miR-188-5p, -877-5p	miR-877-5p aCD vs iCD: miR-140-5p, miR-145-5p, -18a-5p, -128-5p	miR-127-3p, -491-5p, -18a-5p, -145-5p, let-7b, -185-5p, -29c-5p, -19b-3p, -20b-5p, -106a-5p, -17-5p, -222-5p, -135a-3p are common in CD and UC miR-877-5p has role in disease remission	TaqMan human miRNA array RT-qPCR	Peripheral blood and Serum	aCD/9 iCD/9 CO/33	Italy	(38)
miR-34b-3p, -142-5p, -205-5p, -424-5p, -885-5p CD vs UC: miR-656-3p, -744-5p, -1908-5p miR-34b-3p, -484-5p, -574-5p common in both CD and UC	miR-570-3p, -1301-3p	miR-205-5p targets LRRK2, SHIP2/INPPL1, ZEB1, E2F1, ERBB3 and miR-142-5p targets NFE2L2/NRF2 and miR-424-5p targets MYB, CUL2, PU.1 which are either IBD-risk loci or IBD-related known genes	Microarray	Peripheral blood	CD/40 CO/38	Germany	(53)
miR-200c-3p, -181a-2-3p, and -125a-5p	miR-369-3p, -376a-5p, -376c-5p, -411-3p, -411-5p, and mmu-miR-379-5p	Validation cohort: Only miR-16 was significantly downregulated in patients (fold change 0.83, P=0.02).	OpenArray miRNA profiling RT-qPCR	Plasma	CD/6 CO/6 Validation CD/102	Denmark	(61)
miR-595-5p, -1246-5p, -142-5p, -143-5p aCD vs iCD: miR-1246-5p, -595-5p and -142-5p		Validation cohort: Only miR-1246-5p, -142-5p and -143-5p were upregulated and only miR-143-5p is significant. UC vs CD: miR-16-5p	Microarray RT-qPCR	Serum	CD/57 CO/58 Validation CD/10 CO/10	New Zealand	(52)
miR-101-5p and -375-3p	miR-21-5p, -31-5p, -146a-5p, and -155-5p	miR-101-5p in CD patients’ saliva was significantly upregulated.	Microarray RT-qPCR	Peripheral blood	CD /42 CO/35	USA	(31)
miR-30e-5p	miR-1183-5p, -1827-5p, -1286-5p, -504-5p, -188-5p, -574-5p, -192-5p, -149-5p, and -378e-5p	Downregulated miR-1286 and miR-1273d-5p correlated with CD disease activity higher than C-reactive protein and calprotectin	Nanostring nCounter	Serum	aCD/21 iCD/24 CO/21	USA	(60)
miR-223-3p		2.2-fold upregulation in CD 2.8-fold upregulation in UC miR-223-3p has higher Spearman r value in IBD detection than hCRP and ESR.	RT-qPCR	Serum	CD/50 CO/50	China	(3)
miR-631-5p, -4521-5p, -562-5p, -766-3p, -302b-3p, -423-3p, -484-5p, -4707-3p, -483-3p, -4516-5p, -665-5p, -1260b-5p, -2117-5p, -216b-5p, -296-5p, -27b-3p, -188-3p, -770-5p, -1233-3p, -4755-5p, -627-3p, -767-3p, -339-5p aCD and iCD vs CO: miR-1268a-5p, -1297-5p, -1909-3p, -197-3p, -197-5p, -410-3p, -936-5p, -542-5p, -549a-5p, -603-5p, -874-3p, -92a-3p, -933-5p, -941-5p		miR-874-3p targets ATG16L1 and reduces its expression and dysregulates autophagy by a reduction of LC3 in vitro Upregulated in iCD vs CO: miR-548g-3p, -4536-5p, -4448-5p, -30a-3p, -548q-5p, -4461-5p, -133a-3p, -597-5p, -619-3p, -644a-5p	NanoString	Peripheral blood mononuclear cells	aCD/35 iCD/10 UC/46 CO/39	Canada	(59)
aCD and iCD vs CO: miR-106a-5p and -362-3p			RT-qPCR	Peripheral blood	aCD/22 iCD/10 CO/32	Iran	(55)
miR-16-5p, -21-5p and -223-3p		Upregulated miRs were detected in both IBD type, but were higher in CD No significant miR-155-5p expression In remission group miRNAs expression is disease activity dependent	RT-qPCR	Serum	CD/35 CO/20 Remission: CD/15	Germany	(50)

All miRNAs are from comparison between the disease and healthy individual, unless otherwise stated.

aCD, active CD; iCD, inactive CD; CO, Control; N, Numbers per Group.

### Computational Meta-Analysis of Publicly Available High Throughput Studies

In addition to the literature curation, we also performed a meta-analysis of publicly available high throughput studies (microarray and RNA-Seq), including 3 UC (27, 62, 63) and 4 CD (42, 47, 62, 64) patient cohorts (**Table 1**). All included studies contained expression profiling at the level of the intestinal mucosa (colon or ileum). We combined the results of differential expression analysis between the UC or CD and the control group from each study as described in the supplementary section. The three UC datasets are consistent with each other, with most differentially expressed miRNAs being changed in the same direction, in contrast to the CD datasets, where many miRNAs are differentially expressed in opposite directions between the datasets (**Supplementary Figure 1**). The higher heterogeneity observed in the expression profiles from CD patients might be consistent with the more heterogeneous nature of CD compared to UC. There might also be other explanations, including different patients’ demographics, different sample handling, and data generation in different labs.

We obtained a final set of 158 miRNAs consistently differentially expressed between UC patients and controls and 69 miRNAs between CD patients and controls (p-value < 0.05 in at least two datasets and a global adjusted combined logit p-value < 0.05) and consistent in the direction of regulation across all datasets (**Supplementary Files 1, 2** and **Supplementary Figure 2**).

The meta-analysis confirms most of the literature-curated miRNAs and at the same time provides dozens of other miRNAs not previously reported in UC or CD mucosa (e.g., miR-378a-3p, miR-191-5p, miR-92a-3p in UC; miR-30e-5p, miR-26b-5p, let-7f-5p, let-7g-5p, in CD; miR-146b-5p, miR-30d-5p, miR-148a-3p, miR-151a-5p in both UC and CD). In addition, few miRNAs showed different or no significant differential regulation compared to what was found in the literature, including miR-142-3p (30, 32, 45) in CD, which in literature curation showed to be constantly upregulated, while in the meta-analysis, it was constantly downregulated.

Moreover, miRNAs reported in the literature are predominantly upregulated (specifically for UC); however, the meta-analysis indicates an almost equal number of up- and downregulated miRNAs. This might be ascribed to the ease/bias of validation for the upregulated miRNAs for diagnostic purposes with available techniques. Furthermore, the downregulated miRNAs showed a higher average expression, possibly indicating a more substantial functional role of these miRNAs (65) (**Supplementary Figure 3**).

One of the studies (GSE89667) contained both UC and CD cohorts (62), and we used the UC versus CD comparison (adjusted p-value < 0.05), in conjunction with the results of the meta-analysis, to find a set of 18 miRNAs differentially expressed between UC and CD. Among these, e.g., miR-29a-3p, miR-155-5p, or miR-454-3p are upregulated in UC compared to CD, while miR-28-3p, miR-378a-5p or miR-422a are downregulated in UC compared to CD (**Supplementary Data, Sheet 3**).

### Overlap of Colon and Blood miRNAs in UC and CD

**Figure 1:**
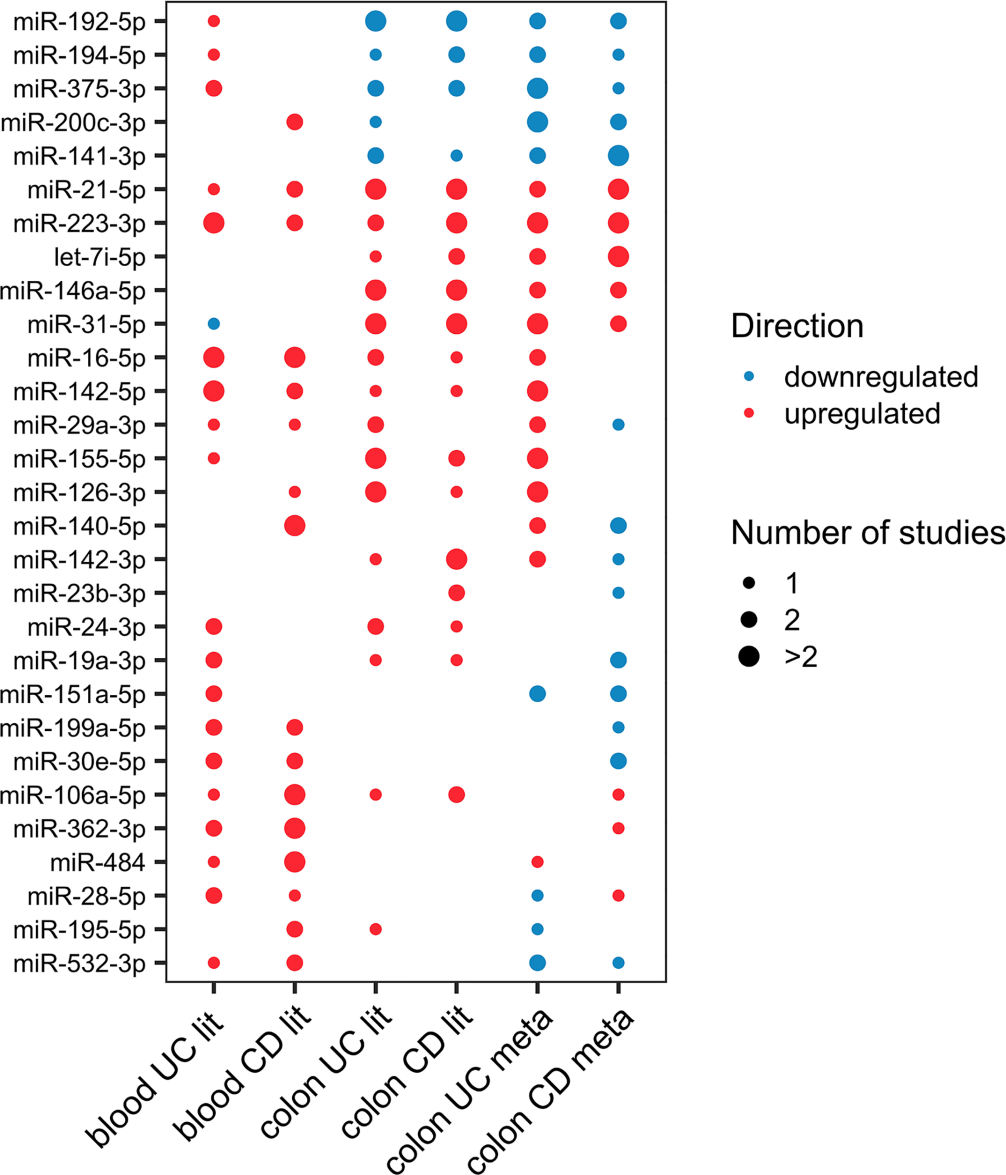
Dot-plot of the 29 differentially expressed miRNAs (at least two studies) in either colon or blood of UC or CD from literature. The node size represents the number of studies, and the node color corresponds to the expression statues, where red means upregulation and blue means downregulation.

There is great potential in identifying disease-specific miRNAs for diagnosis, progression, and therapeutic response. Consistently differentially expressed miRNAs in the colon and blood may have the highest clinical potential. From literature curation, 29 miRNAs were consistently differentially expressed in at least two studies in colon or blood of UC or CD (**Figure 1**).

#### Ulcerative Colitis

From the miRNAs with consistent differential regulation in at least two independent studies, miR-223-3p, miR-16-5p, and miR-24-3p showed upregulation in both mucosa and blood of UC patients compared with healthy individuals. miR-21-5p and miR-146a-5p were also shown to be differentially expressed in both tissues. However, the blood data for these two miRNAs were inconsistent. Considering only one study, 18 miRNAs were commonly differentially expressed in both tissues (**Supplementary Data, Sheet 4**).

#### Crohn’s Disease

From the miRNAs with consistent differential regulation in at least two independent studies in CD miR-223-3p and miR-21-5p showed upregulation in both mucosa and blood of patients compared with healthy individuals. miR-192-5p was also common and frequently downregulated in the mucosa; however, since the data for this miRNA in the blood is inconsistent, it was excluded. Finally, considering miRNAs differentially expressed in only one study, 13 miRNAs were shown to be commonly differentially expressed in both tissues (**Supplementary Data, Sheet 5**).

### UC and CD miRNA Profile Similarities and Differences

Even the most experienced clinicians have problems in the initial diagnosis of IBD and stratifying its subtypes. Stratifying UC and CD has always been a challenge ascribed to their overlapping features. Although these IBD subtypes have common characteristics, significant genetic and clinical differences exist. Consequently, different transcriptome profiles, specifically distinct miRNAs signatures, might improve IBD subtype classification.

#### Colon

Many studies compared individuals with and without the diseases to stratify UC and CD based on mucosa biopsy miRNA signature (30–32, 34, 36, 38). Considering miRNAs validated to be differentially expressed in at least two studies in both UC and CD mucosa, miR-21-5p, miR-31-5p, miR-146a-5p, miR-223-3p showed to be commonly up- and miR-192-5p and miR-375-3p downregulated in both phenotypes.

Furthermore, considering miRNAs with consistent differential regulation in at least two independent studies, miR-155-5p, miR-126-3p, miR-29a-3p, miR-141-3p, miR-16-5p and miR-24-3p showed to be differentially expressed mainly in UC mucosa, while miR-142-3p, miR-150-5p, let-7i-5p, miR-23b-3p, miR-19b-3p, miR-215-5p, miR-629-5p, miR-194-5p and miR-106a-5p showed to be more frequently differentially expressed in CD mucosa.

To confirm the above observation, these miRNAs (from at least two studies) were more intersected against the literature miRNA lists, this time one study and more. The comparison showed that miR-29a-3p is only reported as significantly differentially expressed (SDE) in UC, and miR-23b-3p is only reported as SDE in CD. Moreover, the results for miR-150-5p and miR-215-5p were inconsistent.

#### Blood

Similar attempts to stratify UC and CD based on the blood miRNA profile of patients versus healthy individuals were made (3, 31, 38, 49, 50, 52–55). Considering frequently differentially expressed miRNAs in UC and CD blood, miR-223-3p, miR-142-5p, miR-16-5p, miR-199a-5p, miR-30e-5p, miR-362-3p were significantly differentially upregulated and were common between both phenotypes and thus could be considered as IBD biomarkers.

Furthermore, considering miRNAs with consistent differential regulation in at least two independent studies, miR-146a-5p, miR-150-5p, miR-151a-5p, miR-188-5p, miR-199a-3p, miR-19a-3p, miR-24-3p, miR-28-5p showed to be mainly differentially expressed in UC. miR-484-5p, miR-106a-5p, miR-574-5p, miR-532-3p, miR-200c-3p, miR-195-5p, miR-192-5p, miR-140-5p showed to be more frequently differentially expressed in CD blood.

To confirm this observation, these differentially expressed miRNAs (from at least two studies) in each phenotype were once more intersected against the literature miRNA lists, this time one study and more. The results showed miR-146a-5p, miR-150-5p, miR-151a-5p, miR-199a-3p, miR-19a-3p, miR-24-3p were only SDE in UC. The results for miR-150-5p and miR-199a-3p were inconsistent. Furthermore, miR-200c-3p, miR-195-5p, and miR-140-5p showed only SDE in CD.

### Most Relevant Differentially Expressed miRNAs

**Figure 2:**
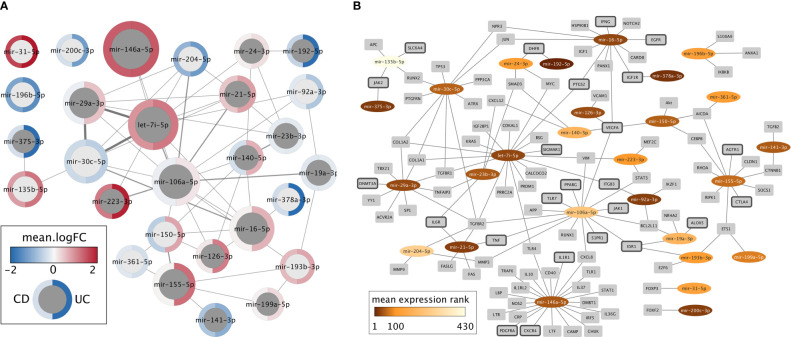
Network representations of the 28 miRNAs with at least one experimentally determined target known to be related to IBD. **(A)** Network of miRNAs only. Dark gray nodes represent miRNAs detected by literature curation, while light gray nodes were not identified in the literature, but only in the meta-analysis. The size of each miRNA node corresponds to the number of IBD targets this miRNA has, and the width of the edges represents the number of shared IBD targets. The mean logFC of each miRNA, according to the meta-analysis, is shown for CD (left) and UC (right) using a blue-white-red gradient on the node border. **(B)** Network of miRNAs (oval nodes) and their target genes (rectangle nodes). miRNAs are colored based on their mean expression rank. Target genes that code for proteins with a clinically approved drug according to the Pharos database are highlighted by dark gray node border color.

To develop miRNA-based novel diagnostics and therapeutics for IBD, it is vital to understand the miRNAs expression changes in correlation with the disease phenotype, underlying mechanisms that regulate miRNAs, the target genes, and their interplay. Despite the heterogeneity of differentially expressed miRNAs in IBD, 66 miRNAs were identified from literature curation and meta-analysis as relevant candidates for diagnostic or therapeutic purposes that might also represent causative agents in disease development (Supplementary data, Sheet 6). For this set of miRNAs, we extracted “experimentally observed targets” from QIAGEN Ingenuity Pathway Analysis (IPA) software program v70750971 (66) and intersected these targets with genes related to IBD extracted from IPA and literature (Supplementary data, Sheet 7). This resulting list of 28 miRNAs with at least one IBD target was visualized in Cytoscape (67) (**Figure 2**). In the following, we discuss most of these miRNAs in more detail.

**Let-7i-5p:** Let-7i-5p is the regulator of TLR4, which is important in cytokine-mediated responses and a regulator of IL-6 (68). In THP-1 cells transfected with let-7i-5p mimics, both mRNA and protein levels of TLR4 showed downregulation (69). Let-7i-5p seems to assist cells in resetting their protein profile in response to external stimuli in allergic inflammation; the exact mechanism is not yet clear (70). Let-7i-5p regulates collagens, IL-6, TGF-βR1, IGF-1, and caspase-3 as primary regulators of inflammation, fibrosis, hypertrophy, and apoptosis (68).

**miR-16-5p:** miR-16-5p in the colonic UC mucosa partly regulates the inflammatory responses through negative regulation of A2aAR (NF-κB inhibitor) expression. miR-16-5p mimics transfection in colonic epithelial cells, demonstrated to increase nuclear translocation of NF-κB p65 protein and thus increase the expression of IFN-γ and IL-8 as important pro-inflammatory cytokines (71).

**miR-19a-3p:** Serum miRNA profiling of CD patients with and without strictures showed miR-19a-3p and miR-19b-3p as potential pathogenic markers (72). Low levels of miR-19a-3p and miR-19b-3p were strongly correlated with stricturing CD and independent of site, gender, age, disease duration, and activity (72). Moreover, it has been reported that miR-19a-3p decreases the SOCS3 expression, which consequently enhances IFN-α and IL-6 signal transduction (73).

**miR-21-5p:** miR-21-5p showed an essential role in colon epithelial cell hemostasis (74), adaptive immune responses (75), cytokine regulation (76), and IBD-related complications (77). It has been demonstrated that in response to epithelial damage, miR‐21-5p causes more intestinal permeability. Transfection of miR-21-5p mimics resulted in the loss of tight junction proteins, increased barrier permeability (74), and decreased CD3 and CD68 positive cells in the UC mouse model (78). The miR-21-5p knockout mice model also showed high resistance to dextran sulfate sodium (DSS) induced colitis, suggesting the pro-apoptotic effect of this miRNA. miR-21-5p also demonstrated an essential role in adaptive immune responses in T-cell function, with the highest detected expression in effector T cells, memory T cells, and the lowest in naive T cells (75). miR-21-5p has a regulatory role in innate immunity and is involved in TLR4 activation and monocyte differentiation. It is also induced by danger signals, such as activators of NF-kB in a negative feedback loop, to prevent damage (79). miR-21-5p is associated with disease activity in UC patients (80). Moreover, this miRNA regulates IL-12 release from dendritic cells and macrophages by targeting the IL-12p35 receptor (76). On the other hand, the association of this miRNA with irreversible IBD fibrosis and its increased level was observed in serum of humans with significant fibrosis (77) and development of dysplasia (81). It is noteworthy that several cellular injury models have shown to be TNF-α dependent with subsequent miR-21-5p induction (77, 82).

**miR-23b-3p:** miR-23b-3p represses autoimmune inflammation by suppressing (IL-17, TNF-α, IL-1β)-induced NF-κB activation, inflammatory cytokine expression by targeting TGF-β-activated kinase 1/MAP3K7 binding protein 2 (TAB2), TAB3 and inhibitor of NF-κB kinase subunit α. Conversely, IL-17 contributes to autoimmune pathogenesis by suppressing miR-23b-3p expression and promoting proinflammatory cytokine expression (83).

**miR-24-3p:** miR-24-3p is reported to be involved in T cells proliferation, differentiation, and immune response (84). It is also reported that miR-24-3p targets Bcl-2 and PAK4 as prosurvival genes, thus, inducing cell death (85). Overexpression of PMS2L2 prompts miR-24-3p gene methylation, resulting in its inhibition. PMS2L2 overexpression, stimulated by LPS, is shown to promote Bcl-2 expression and to inhibit Bax, cleaved-caspase-3, and cleaved-caspase-9 expressions (86). Furthermore, miR-24-3p regulates the processing of latent TGF-β1 release by furin targeting (87). miR-24-3p is reported to downregulate not only TGF-β1, furin, and TNFAIP3 (88).

**miR-28-5p:** miR-28-5p are shown to be involved in cell proliferation, migration, invasion, and epithelial to mesenchymal transition (EMT) (89). miR-28-5p can silence PD1 genes and regulate the PD1, Foxp3 positive and TIM3, Foxp3 positive, exhaustive Treg cells (90).

**miR-29a-3p:** miR-29a-3p has a seven-nucleotide wide binding site on the 3’UTR of the MCL-1 gene and could be involved in the UC pathogenesis through regulating this gene. Mcl-1 gene knockout is shown to cause apoptosis in the colonic epithelial HT29 cells (91). Increased expression of miR-29a-3p in the colon tissues of patients with irritable bowel syndrome increased intestinal membrane permeability, regulating the GLUL gene (92). Moreover, miR-29a-39 is reported to regulate pro-inflammatory cytokine secretion and scavenger receptor expression *via* LPL targeting in ox LDL-stimulated dendritic cells (93).

**miR-30d-5p and miR-30c-5p:** Oral administration of miR-30d-5p mimic ameliorates experimental autoimmune encephalomyelitis (EAE) through expansion of Tregs. In *Akkermansia muciniphila*, miR-30d-5p regulates lactase expression and increases *Akkermansia* abundance in the gut. Consequently, *Akkermansia* increases Tregs to suppress EAE symptoms (94). miR-30c-5p regulates ATG5 expression by targeting the 3’UTR (95). The inverse correlation between miR-30c-5p and ATG5 is not only observed in CD patients and intestinal epithelial T84 cells infected with the adherent-invasive *Escherichia coli* (AIEC) (95). The NF-κB pathway was shown to be activated in AIEC infected T84 cells, which induced the up-regulation of miR-30c-5p and consequently inhibited the ATG5 expression (95). It has further been reported that the autophagic activity inhibition by miR-30c-5p increased AIEC persistence within T84 cells and increased pro-inflammatory cytokines production (95). miR-30c-5p is also believed to regulate Th17 cells differentiation by targeting its negative regulators such as SMAD2, SMAD4, TGFβR2, SOCS3, FOXO3, and TSC1 (96). Thus, their differential regulation might cause an increase or decrease in Th17 cell numbers. ETS1, BCL6, and STAT1 are also among the important targets of miR-30c-5p (96).

**miR-31-5p:** miR-31-5p showed a gradual upregulation from normal to IBD conditions and seemed to target FIH-1, the inhibitor of HIF-1α protein (97). Also, in psoriasis, miR-31-5p inhibition in keratinocytes was shown to suppress NF-kB–driven promoter-luciferase activity and production of IL-1β, CXCL1, and CXCL5. miR-31-5p regulates these cytokine and chemokine expressions in endothelial cells and attracts leukocytes *via* STK40 as its primary target (98). miR-31-5p also targets Gprc5a, which is shown to be a critical regulator for peripherally derived regulatory T cells generation. miR-31-5p conditional deletion enhances induction of these regulatory T cells and decreases the severity of experimental autoimmune encephalomyelitis (99). IL-13 is a necessary type-2 T-helper cytokine, controlling epithelium function through the IL-13 receptor -A1. It has been shown that the transfection of miR-31-5p and miR-155-5p mimics reduces the expression of the IL-13 receptor, increases and blocks the phosphorylation of STAT6, and the expression of SOCS1 and CCL26 in the gut epithelium cell line, and therefore may contribute to disease aggravation (33). Furthermore, miR-31-5p is differentially expressed in post-ablation epithelium with increased barrier permeability (100).

**miR-106a-5p:** Serum level of miR-106a-5p in both CD and UC patients correlates with disease severity (55). Upon T cell activation, while most miRNAs are downregulated, miR-106a-5p is upregulated (101). In addition, in macrophages, miR-106a-5p can regulate SIRPα synthesis and, therefore, SIRPα-mediated inflammatory responses (102). miR-106a-5p deficiency showed to promote Treg induction IL-10 production and attenuate adoptive transfer colitis in T cell restricted deficiency (103). In non-colonic cell lines, miR-106a-5p regulates IL-10 expression (103). Moreover, in CD4+ T cells, miR-106a-5p miRNA family deletion also attenuated the inflammation in lymphopenic recipients. Global knock-out of miR-106a-5p was also shown to attenuate chronic murine ileitis (104). TGFβ appears to suppress miR-106a under physiological conditions to aid Treg induction. TNFα, on the other hand, appears to drive upregulation of miR-106a-5p under inflammatory conditions through NF-κB-dependent induction of the miR-106a-5p promoter, resulting in temporary suppression of normal immune regulation (104).

**miR-126-3p:** IκBα as the inhibitor of NF-κB was shown to be markedly decreased in active UC tissues (105). miR-126-3p and IκBα expression are inversely correlated in patients with active UC. miR-126-3p is shown to contribute to UC pathogenesis through binding to the 3′- UTR of IκBα and inhibiting the NF-κB signaling pathway (105). Anti-inflammatory activities of the red wine polyphenols were partly mediated through miR-126-3p induction (106). Polyphenolic red wine extract (WE) inhibited inflammation in LPS-stimulated human colon-derived CCD-18Co cells by inhibiting NF-κB and down-regulating pro-inflammatory agents, including TNF-α, IL-6, and CAMs. miR-126-3p was upregulated upon WE treatment in these cells, and NF-κB and VCAM-1 showed downregulation (107). VCAM-1 is one of the miR-126-3p targets (108). miR-126-3p knockdown is reported to up-regulate the PIK3R2 in CD8+ T cells (109) and alter the PI3K/Akt pathway activation responsible for regulatory T cells reduced induction and suppressive function (109). Moreover, IκB, an inhibitor of NFκB, is another target of miR-126-3p (109).

**miR-140-5p:** miR-140-5p is shown to downregulate TLR4 by being directly bound to its 3′UTR, which inhibits inflammatory cytokines secretion (110). Moreover, it has been demonstrated that miR-140-5p inhibited IL-6 and IL-8 secretion by regulating TLR4 expression (110).

**miR-141-3p:** miR-141-3p is aberrantly expressed in IBD and other autoimmune diseases, including lupus and psoriasis (111, 112). miR-141-3p targets CXCL12β (113), an epithelial cell-expressed chemokine whose inverse correlation with miR-141 is shown in the inflammation. Therefore, it is suggested that targeting CXCL12β by miR-141-3p might influence inflammatory cell trafficking into the inflamed sites. Thus, inhibiting colonic CXCL12β expression and blocking immune cell recruitment might be valuable for the CD treatment (113). miR-141-3p is also reported to suppress STAT4, thus, inhibiting inflammatory factors (114). miR-141-3p upregulation reduces the IL-1β, TNF-α, and IL-6 levels, consequently attenuating the chronic inflammatory pain severity (115). Furthermore, during Th17 cell induction, miR-141-3p expression is reported to be significantly upregulated (116). miR-141-3p can also exert protective effects on cell damage (114). It is also reported that miR-141-3p alleviates LPS-induced intestinal epithelial cell injury by inhibiting RIPK1-mediated necroptosis and inflammation (117).

**miR-142-5p(-3p):** In thymically derived Tregs, miR-142-5p is the predominant isoform. Tregs limit the development of autoimmunity by suppressing self-reactive peripheral T effector cell responses (118). miR-142-5p is shown to target SMAD3, CYR61 (119), and PD-L1 (120). Regulation of PD-L1 expression is through binding to its UTR and inversely correlated with miR-142-5p (121). TNF-α, IFN-γ, and IL-10, as prominent players in the immune response, are related to the PD-L1/PD-1 pathway. It has been shown that miR-142-5p overexpression results in TNF-α and IFN-γ upregulation and IL-10 downregulation (121). ATG16L1, as one of the most commonly detected genetic variations in CD patients, is predicted to be the target of miR-142-3p (122, 123). miR-142-3p negatively regulates ATG16L1 in CD colon epithelial cells. Upregulation of miR-142-3p reduced the autophagic activity of thymic-derived regulatory T cells by decreasing the expression of ATG16L1 (124). miR-142-3p binds directly to KDM6A (a lysine demethylase), resulting in the demethylation of H3K27me3, an epigenetic modification to the DNA packaging protein Histone H3. This, in turn, upregulates the expression of the anti-apoptotic protein Bcl-2. It has also been shown that antagomir-mediated knockdown of miR-142-3p can affect the induced regulatory T cells regulatory function, cytokine expression, and apoptosis through Foxp3 expression (125). Moreover, downregulation of miR-142-3p in macrophages of aged mice contributed to IL-6-associated aging disorders and consequently age-related inflammatory diseases (126).

**miR-146a-5p and miR-146b-5p(-3p):** miR-146a-5p has previously been shown to regulate the innate immune responses and TNF-α pathway in skin inflammation (127). miR-146a-5p deficient mice also develop immune disorders (128). In IBD, this miRNA regulates NOD2 derived gut inflammation and promotes proinflammatory cytokines released from activated macrophages (129). Moreover, upregulation of miR-146a-5p in monocytes in response to LPS resulted in the downregulation of TLR4 signaling pathway downstream genes (130). On the other hand, in mouse colitis, miR-146b-5p overexpression was shown to alleviate intestinal injury *via* NF-κB activation, epithelial barrier function improvement, and increased survival rate (131). miR-146b-5p seems to up-regulate NFκB *via* siah2 suppressing. Siah2 prompts TRAF proteins ubiquitination which is upstream of NFκB (131). miR-146b-3p, another member of the miR-146 family, is shown to negatively regulate lipid kinase PI3Kγ in (132), suppress proinflammatory ADA2, and block TNF-α secretion (133). Furthermore, miRNA-146b-3p expression is significantly downregulated by increased STAT3 activation (134).

**miR-149-5p:** Through targeting MyD88, miR-149-5p negatively regulates TLR triggered inflammatory cytokine production (135). MyD88 is involved in the TLR/NF-κB pathway. miR-149-5p is also associated with an increased IBD risk in the Chinese population (136).

**miR-150-5p:** miR-150-5p is proposed as one of the primary regulators of immune diseases (137), mainly through inhibiting inflammatory cytokines including IL-6, IL-1β, and TNF-α (138). It is also reported that the miR-150-5p upregulation in immune cells promotes the proliferation and maturation of myeloid cells and lymphocytes (139). c-Myb, a target of miR-150-5p, is reported to be significantly downregulated in UC patients’ colon and DSS-treated mice. miR-150-5p overexpression is reported to enhance apoptosis through targeting c-Myb, which damages the intestinal epithelial barrier (140).

**miR-155-5p:** miR-155-5p has shown a central regulatory role in innate and acquired immune systems. miR-155-5p is expressed in response to inflammatory mediators such as LPS, TLR ligands, and IFN-β and is induced in antigen-presenting cells, including plasmacytoid dendritic cells and macrophages. It has also been found that antigen-stimulated B and T cells induce miR-155-5p expression (141). Moreover, SOCS1, a negative regulator for activation of LPS-induced macrophage, JAK/STAT signal pathway, and antigen presentation by dendritic cells, is one of the main targets of miR-155-5p (142). In addition, Anti-miR-155-5p has been reported to suppress G-CSF, a regulator of granulopoiesis produced by macrophages during acute inflammation (143). Increasing expression of the level of this miRNA has also been shown in other inflammatory disorders, such as rheumatoid arthritis (144), atopic dermatitis (145), and multiple sclerosis (146). In addition, it has been reported that miR-155-5p is an oncogene (147).

**miR-192-5p:** miR-192-5p is shown to target MIP-2α (CXCL2), a CXC chemokine expressed by epithelial cells and essential in murine and human IBD. miR-192-5p is downregulated in inactive UC and demonstrated an inverse correlation with MIP2-α. miR-192-5p mimic was reported to inhibit MIP2-α induced MIP-2a expression (26). miR-192-5p is induced by TGF-β and TNF-α (26, 39) and regulates the collagen and chemokine expression, which are critical in inflammation and fibrosis (148). miR-192-5p is also identified as a tumor suppressor that can induce cell cycle arrest (149).

**miR-193b-3p:** miR-193b-3p differential regulation has been detected in several autoimmune diseases (150), mainly through inflammatory chemokines regulation (151). miR-193b-3p has been shown to target TGF-β2 and TGFBR3 3′-untranslated regions (152) and contribute to Th17 cells differentiation by inhibiting the negative regulators of Th17 differentiation expression and possibly through regulating TLR and Notch signaling pathways. Thus, suggesting the possible involvement of miR-193b-3p in the inflammatory response and Th17 function (153).

**miR-194-5p:** miR-194-5p is abundant in intestinal epithelial cells (39) and is shown to regulate the MAP4K4/c-Jun/MDM2 signaling pathway (154). Overexpression of miR-194-5p in the liver mesenchymal cells reduced the N-cadherin (155). In the Caco-2 intestinal epithelial cell model, HNF-1α induced miR-194-5p suggesting the influence on epithelial cell differentiation (156).

**miR-195-5p:** miR-195-5p is shown to correlate with IBD severity. An increase in miR-195-5p level can decrease c-Jun and p65 expression. Instead, miR-195-5p decreased expression increases Smad7 expression and consequently p65 and the AP-1 upregulation, which might explain the steroid resistance mechanism in some UC patients (157). miR-195-5p overexpression was shown to reduce M1-like macrophage polarization. miR-195-5p levels are reported as upregulated in M2c macrophages. LPS and IFN-γ stimulated THP-1 macrophages had reduced TLR2 levels following miR-195-5p overexpression. miR-195-5p also significantly decreased IL-1β, IL-6, and TNF-α levels in M1-stimulated macrophage supernatant cultures. In addition, levels of phosphorylated forms of p54 JNK, p46 JNK and p38 MAPK were shown to decrease by adding miR-195-5p in M1 macrophages upon stimulation. Altogether it seems like miR-195-5p is involved in macrophage polarization by inhibiting TLR2 inflammatory pathway mediators (158).

**miR-199a-5p:** miR-199a-5p showed significant upregulation in blood from UC patients compared with healthy controls (54). miR-199a-5p seems to suppress HIF-1α and SIRT1 and play a role in Treg cell differentiation by inhibiting genes involved in Th17 differentiation while activating others in Treg development (159, 160). RORγt is a lineage-specific transcription factor for Th17 differentiation. In multiple sclerosis, RORγt expression, a predicted target for miR-199a-5p (using miRWalk, miRTarBase, DIANA miRPath, UniGene), showed a significantly higher level in the relapsing phase versus remitting phase. This is consistent with the upregulation of miR-199a-5p, which correlates with lower Th17 cells and lower expression of RORγt in remitting phase (96). It has also been reported that miR-199-5p targets the activin A receptor type 1B gene that causes decreased CCAAT/enhancer-binding protein α expression and eventually monocyte/macrophage differentiation inhibition (161).

**miR-200c-3p:** miR-200c-3p plays a role in the FN1 post-transcriptional regulation; hence, EMT triggers by their downregulation (162, 163) most probably by regulating the E-cadherin transcriptional repressors ZEB1 and SIP1 (164). miR-200c-3p is reported to suppress the IL-6, CXCL9, and TNF-α expression (165). IL-6 intensifies inflammation through miR-200c-3p downregulation (166). In a macrophage-like human monocytic cell line exposed to the TLR4 ligand LPS, miR-200c-3p inhibits NF-κB activation in response to a TLR4 agonist. miR-200c-3p is known to regulate the TLR4 signaling efficiency through the MyD88-dependent pathway (167).

**miR-223-3p:** miR-223-3p is shown to be involved in the activation of granulocytes and is overexpressed in naive CD4+ T-lymphocytes (168). Furthermore, the downregulation of miR-223-3p in primary macrophages increased TLR4 and STAT3 basal expression and LPS-stimulated TLR4, STAT3, and NOS2 expression. On the contrary, miR-223-3p mimics treatment in primary macrophages has decreased TLR4 expression while negatively regulating FBXW7 expression, a well-known suppressor of TLR4 signaling. Based on these outcomes, it is concluded that miR-223-3p abundance in macrophages can change macrophage activation and modulate the response to stimuli *via* effects on the TLR4/FBXW7 axis (169). It has also been shown that miR-223-3p mediates the cross-talk between the intestinal barrier and the IL-23 pathway by targeting CLDN8, a claudin protein that constitutes the backbone of the intestinal barrier (170). miR-223-3p has also been used as a biomarker in IBD (3). Thus, the evidence suggests its proinflammatory role and highlights its potential as a RNA biomarker that seems to be conserved between different species. miR-223-3p is also produced by neutrophils and monocytes and acts as a controller of NLRP3 inflammasome activity, regulating the intestine inflammatory process by affecting IL-1β production (171).

**miR-375-3p:** miR-375-3p is reported to be downregulated in the intestinal mucosa of UC and CD patients. TLR4 is one of the main targets of miR-375-3p with inverse correlation. miR-375-3p mediated upregulation of TLR4 induces NF-κB activation, which leads to an increase in pro-inflammatory factors (172). Intestines show a high level of miR-375-3p expression. Cell death, including apoptosis and/or necrosis, results in the miR-375-3p leak from cellular to extracellular space, eventually ending in the blood. Therefore, it is suggested that elevated miR-375-3p in serum may be a predictor of tissue damage (173).

**miR-378a-3p:** miR-378a-3p expression is reported to be inversely correlated with IL-33 expression; IL-33 is a predicted target of miR-378a-3p (174). miR-378a-3p is highly conserved between species, but not IL-33 (175). The miR-378a-3p is located in intron 1 of the PPARGC1B gene that is differentially regulated in UC patients’ intestinal mucosa ^26^. PPARGC1B protein is highly expressed in the intestinal epithelium (176) and is involved in the control of mitogenesis and mitochondrial metabolism (177), energy production, and biogenesis (178). Therefore, it can be concluded that in inflamed mucosa, the miR-378a-3p decrease might reflect a metabolic shift, possibly related to the increment of energy expenditure and ROS overproduction (179).

**miR-424-5p:** miR-424-5p is shown to control monocyte/macrophage differentiation. miR-424-5p expression upregulation is regulated by transcription factor PU.1. When upregulated, miR-424-5p induces monocyte differentiation *via* NFI-A inhibition (180) as its main target.

**miR-532-3p:** miR-532-3p acts as an antagonist for LPS/TNF-α stimulated macrophages by targeting the ASK1/p38 MAPK signaling pathway, thus suppressing the inflammation, which is mediated through this pathway. Thus, it has been suggested as a potential target for treating autoimmune inflammatory diseases (181).

## Concluding Remarks

Early diagnosis and treatment are vital in IBD, as induction of early remission and maintenance can prevent long-term complications and eliminate the need for surgery. However, due to insufficient clinical sensitivity and specificity of current biomarkers and a large population of patients with functional bowel disorders, there is often a delay in the confident diagnosis of IBD and its sub-classification into either UC or CD (182). At the same time, the primary way to overcome IBD is to induce and maintain early remission. Most current IBD diagnostic tests reflect generalized inflammation and do not discriminate between IBD subtypes (182).

Since their discovery, thousands of miRNAs have been identified. Accumulating evidence suggests that specific miRNA expression signatures contribute to the IBD development and progression. Most studies reveal correlations between IBD and differentially expressed miRNAs instead of causal relationships. As discussed above, only a few studies investigate the underlying molecular mechanisms of the disease; thus, the precise function of most miRNAs in IBD has yet to be clarified. Furthermore, there has been a lack of reproducibility between studies, partly ascribed to a lack of standardized study designs and different approaches.

Moreover, many variables differ between studies, including age, sex, various treatment regimens, disease activity level and duration, having different control groups, sampling from different anatomic locations, sampling method, preservation and processing of the samples, and the different criteria for measuring expression fold change and significances (e.g., different FC, log FC, p-value and p-adj criteria). Thus, it is essential to understand the conditions under which a differentially expressed miRNA was discovered. For instance, epigenetic regulations are among the primary factor stimulated by the environment. Stimuli such as diet, lifestyle, work condition, and stress are elements as important as the clinical and technical manifestations of signs of disease. Regardless of these differences, while being aware of them, in this review, we attempted to identify and give an overview of the most frequently differentially expressed miRNAs in colon and blood of both UC and CD across multiple studies from literature and meta-analysis and further described the roles of selected miRNAs in the disease pathogenesis and their connection to IBD.

For biomarker studies, circulating miRNAs (of saliva, serum, urine, plasma, and other body fluids) attracted great interest as non- or semi-invasive clinical biomarkers mainly due to ease of access, stability, conserved structure, and ease of detection by quantitative approaches like real-time PCR. The need for endoscopic examination and invasive sampling of biopsies limit the use of colonic miRNAs as biomarkers. Thus, if a miRNA demonstrates a similar consistent differential regulation in colonic biopsies and blood of the IBD patients compared with healthy control, it can be used as a proper disease biomarker signature. miR-223-3p, in this case, might be an excellent example of such miRNAs. This miRNA is significantly differentially expressed in both UC and CD in blood and tissue biopsies and thus can be considered a reliable IBD biomarker candidate.

Anti-cytokines therapies have been relatively successful; however, not all patients respond to these treatments. As important post-transcriptional gene regulators, miRNAs were shown to contribute to disease aggravation through immune responses, inflammation, mucus barrier, and epithelium function dysregulation; thus, miRNA-based therapy might be developed as a potential therapeutic approach. In this case, miRNAs complementary antisense oligonucleotides or miRNA mimics can be potential therapeutics that abolish or mimic miRNA’s function and, therefore, block inflammatory progression, modulate cytokines or chemokine hemostasis and increase the treatment sensitivity of conventional therapies. As such, miRNAs are used for modulating hypoxia (183, 184) and the inflammatory response by targeting major inflammatory pathways (185–189) and essential molecules, including tight junction proteins that maintain the integrity of the membrane (74, 190, 191).

## Future Perspectives

Although progress has been made towards understanding the role of miRNAs in IBD pathophysiology, many conditions and many more miRNAs remain insufficiently characterized for diagnostic and therapeutic applications, partly as it is still a relatively young field. Also, as a chronic disease with flare-ups and remission, besides comparing disease versus control, it is relevant to look at disease subgroups, e.g., the differences between active/inactive and inflamed/not inflamed intestinal regions. While some studies grouped patients into active UC, inactive UC, inflamed UC, and non-inflamed UC, still further studies are needed to improve our understanding. In addition, it remains to be determined how associations with IBD risk loci might affect miRNA’s expression and the disease phenotype. Moreover, although it has been less focused on, the disease activity index can also be assessed by profiling miRNA specifically at different disease stages while maintaining that miRNA expression is often tissue or pathology specific.

Due to the IBD complexity and the lack of consistency between miRNA signatures, it is difficult to diagnose the disease, identify the subtypes, and monitor the disease status or location using a single or even a panel of miRNAs. Although there is an imperious need for faster ways to validate miRNAs as biomarkers, the sensitivity and specificity of miRNA candidates should be checked in large-scale studies to avoid false positive or false negative diagnosis.

Differentially expressed miRNAs profiling can be a valuable indication of phenotypic changes in IBD, showing an obvious correlation with disease evolution. However, differential expression *per se* does not indicate the ultimate role of the identified miRNAs in disease pathophysiology, as there are complex networks of interaction between miRNAs and their targets that also depend on the cell type, location, and tissue condition. It is noteworthy that many miRNAs might have the same target. Thus, when it comes to the therapeutic interventions using the miRNAs, the main issue is the side effects of miRNA-based drugs that need to be considered in extensive validation studies before miRNAs can enter the market and be incorporated into clinical practice. Also, miRNA expression as measured on high-throughput platforms, e.g., RNA-sequencing, has limitations. For example, if a highly expressed target is downregulated, the expression of the miRNA will appear as increased despite the miRNA being processed at the same rate, i.e., miRNA itself is not directly regulated. Extending miRNA analysis to be “target context-aware” rather than looking at miRNA solely from small RNA-sequencing will likely shed more nuances on to cause and effect of regulated miRNAs and thereby pave the way for considering miRNAs in diseases. Despite the present limitations, we anticipate that miRNAs application and targeting will become routine diagnostic and therapeutic approaches in clinical settings as current techniques evolve rapidly.

## Author Contributions

All authors have made substantial contributions to conception and design, acquisition of data, or analysis and interpretation of data. All authors contributed to the article and approved the submitted version.

## Funding

This work was supported by the Independent Danish Research Foundation, Technology, and Production, grants 4005-00443 and 8020-00300B, the Novo Nordisk Foundation, grant NNF14CC0001, Lundbeck Foundation, grant R303-2018-3148, and the Sehested Hansen foundation. The funders had no role in study design, data collection, analysis, or manuscript preparation.

## Conflict of Interest

The authors declare that the research was conducted in the absence of any commercial or financial relationships that could be construed as a potential conflict of interest.

## Publisher’s Note

All claims expressed in this article are solely those of the authors and do not necessarily represent those of their affiliated organizations, or those of the publisher, the editors and the reviewers. Any product that may be evaluated in this article, or claim that may be made by its manufacturer, is not guaranteed or endorsed by the publisher.
