# Supplementary material for: Differentially Expressed miRNAs in Ulcerative Colitis and Crohn’s Disease
Source: Front Immunol. 2022 Jun 6;13:865777. doi: 10.3389/fimmu.2022.865777 (PMC9208551; doi:10.3389/fimmu.2022.865777)
Supplement: Supplementary file 1 [file DataSheet_1.zip › Supplementary Material/Supplementary Figures.PPTX]

## Slide 1
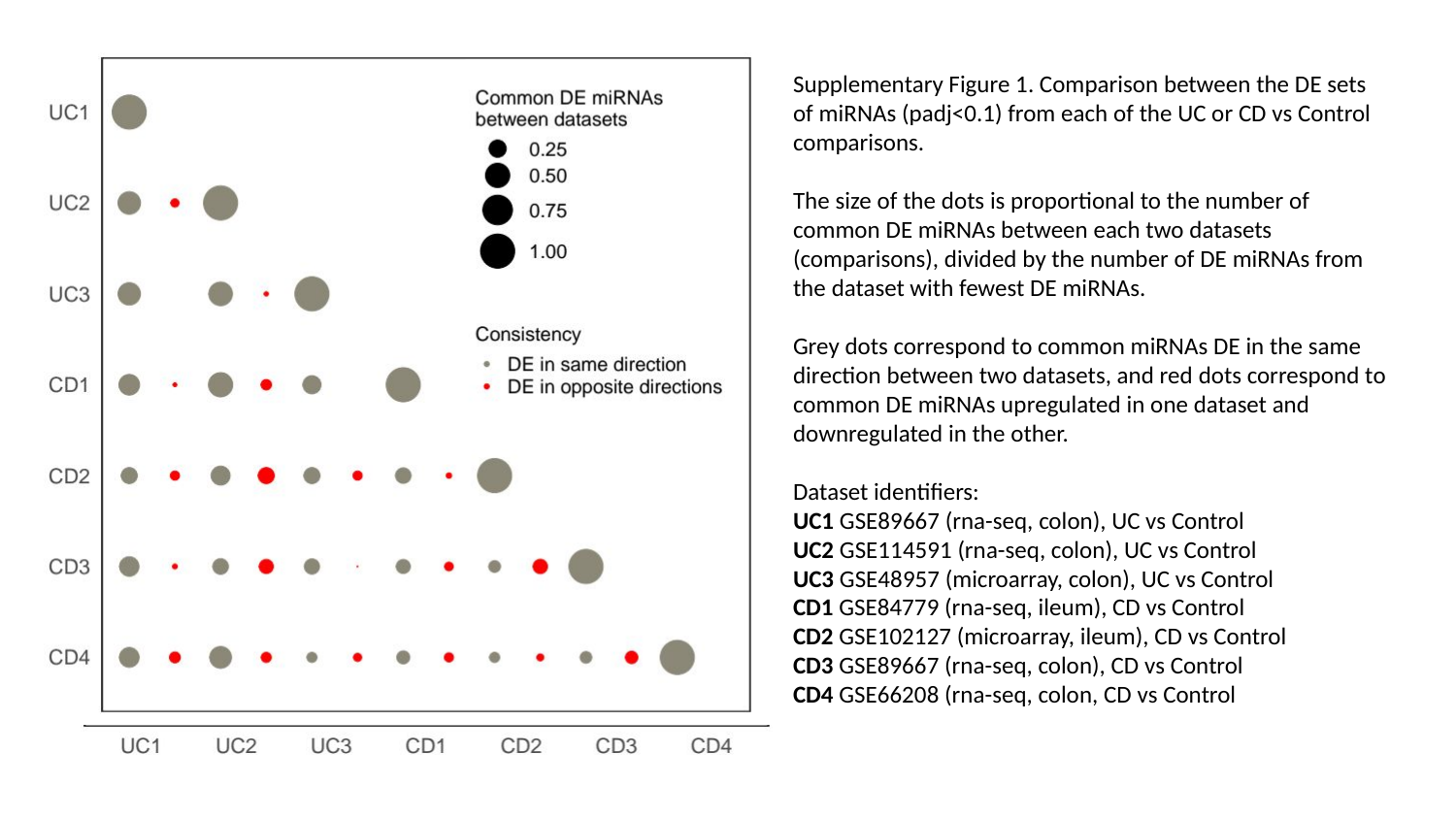

Supplementary Figure 1. Comparison between the DE sets of miRNAs (padj<0.1) from each of the UC or CD vs Control comparisons.
The size of the dots is proportional to the number of common DE miRNAs between each two datasets (comparisons), divided by the number of DE miRNAs from the dataset with fewest DE miRNAs.
Grey dots correspond to common miRNAs DE in the same direction between two datasets, and red dots correspond to common DE miRNAs upregulated in one dataset and downregulated in the other.
Dataset identifiers:
UC1 GSE89667 (rna-seq, colon), UC vs Control
UC2 GSE114591 (rna-seq, colon), UC vs Control
UC3 GSE48957 (microarray, colon), UC vs Control
CD1 GSE84779 (rna-seq, ileum), CD vs Control
CD2 GSE102127 (microarray, ileum), CD vs Control
CD3 GSE89667 (rna-seq, colon), CD vs Control
CD4 GSE66208 (rna-seq, colon, CD vs Control

## Slide 2
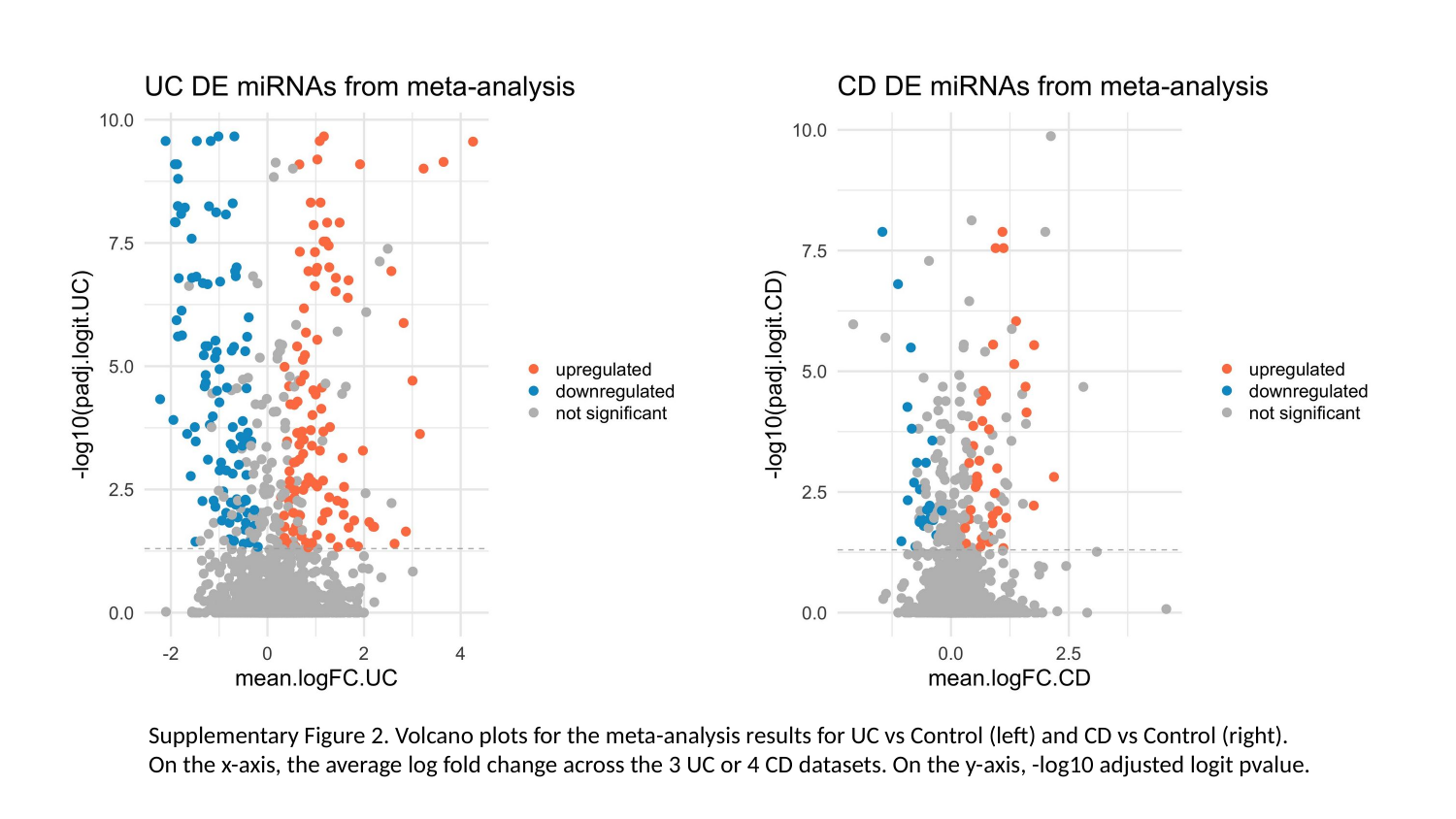

Supplementary Figure 2. Volcano plots for the meta-analysis results for UC vs Control (left) and CD vs Control (right).
On the x-axis, the average log fold change across the 3 UC or 4 CD datasets. On the y-axis, -log10 adjusted logit pvalue.

## Slide 3
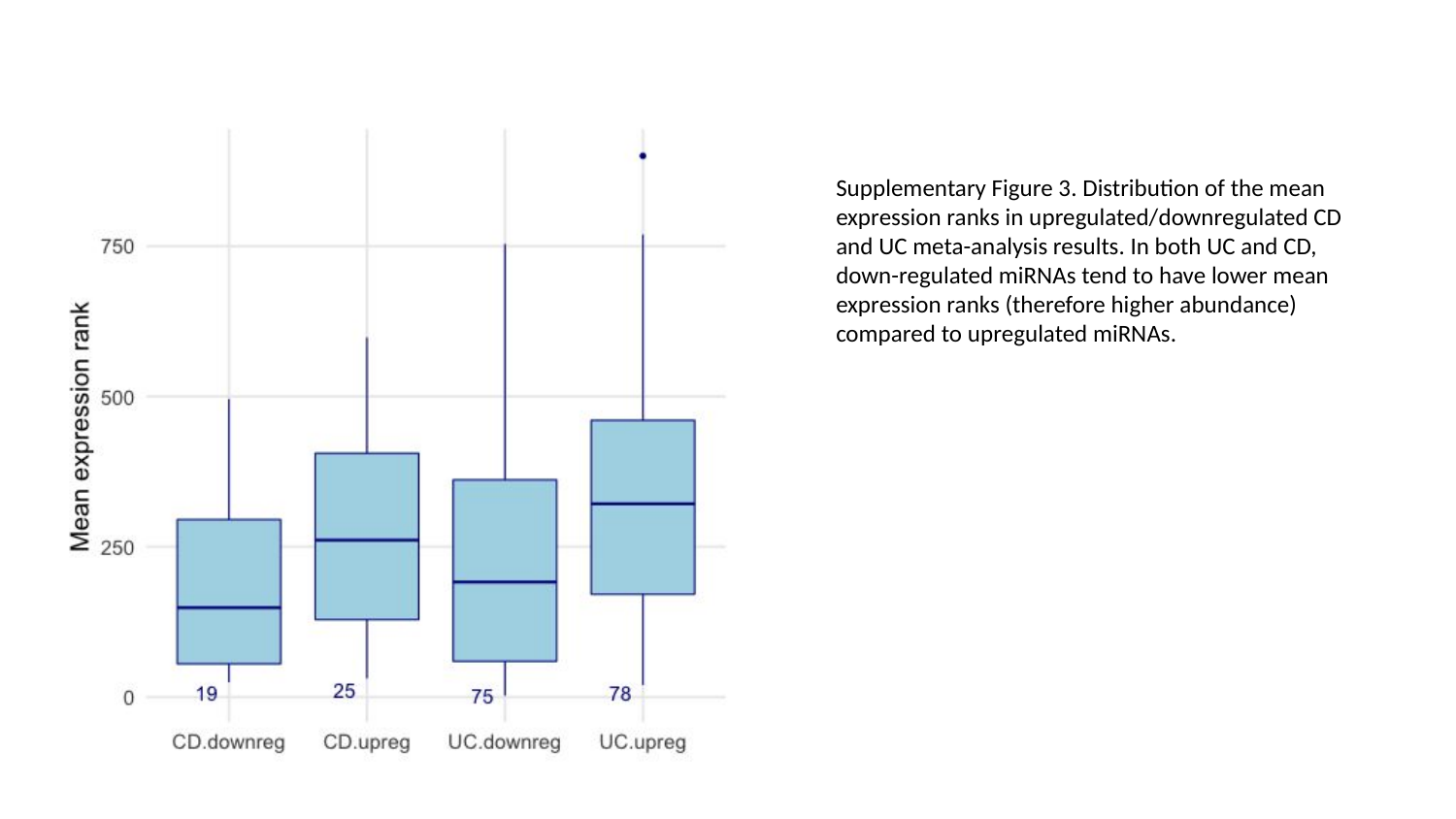

Supplementary Figure 3. Distribution of the mean expression ranks in upregulated/downregulated CD and UC meta-analysis results. In both UC and CD, down-regulated miRNAs tend to have lower mean expression ranks (therefore higher abundance) compared to upregulated miRNAs.
