# Supplementary material for: Differentially Expressed miRNAs in Ulcerative Colitis and Crohn’s Disease
Source: Front Immunol. 2022 Jun 6;13:865777. doi: 10.3389/fimmu.2022.865777 (PMC9208551; doi:10.3389/fimmu.2022.865777)
Supplement: Supplementary file 1 [file DataSheet_1.zip › Supplementary Material/Supplementary Section.docx]

# Supplementary Section

## High throughput datasets analysis

We included six high throughput datasets (4 RNA-Seq and 2 Affymetrix) in the meta-analysis (Supplementary table 1). We considered for inclusion all affymetrix/RNA-Seq datasets comparing expression in UC and/or CD patients with control patients, available on GEO Datasets up to February 2021. Of these, we excluded datasets containing fewer than 3 samples per group or studies pooling samples before sequencing/hybridizing to the array. We re-analyzed the datasets using our own pipelines such that we can make all relevant comparisons between the different conditions. Furthermore, we ensured that the data is processed in a similar fashion and by this made the datasets more comparable.

Supplementary table 1: Publicly available high throughput studies included in our meta-analysis.

| Accession (Ref in main text) | Description | Platform | Method | Samples used |
| --- | --- | --- | --- | --- |
| GSE89667 (62) | UC and CD, colon | RNA-Seq | Illumina HiSeq 2500 | 10 UC, 9 CD, 18 Control |
| GSE48957 (63) | UC, colon | Microarray | Affymetrix Multispecies miRNA-2 Array | 10 UC, 10 Control |
| GSE114591 (64) | UC, colon | RNA-Seq | TruSeq Small RNA Sample Prep Kit V1 | 15 UC (6 non-responders and 9 responders to steroid treatment, samples taken before treatment), 6 Control |
| GSE84779 (65) | CD, ileum | RNA-Seq | Illumina HiSeq 2500 | 10 CD, 12 Control |
| GSE102127 (40) | CD, ileum | Microarray | Affymetrix Multispecies miRNA-2 Array | 20 post-operatory recurrent CD + 7 late CD, 8 Control |
| GSE66208 (43) | CD, colon | RNA-Seq | Illumina HiSeq 2500 | 18 CD, 13 Control |

The RNA-seq datasets were analyzed using the following pipeline. We obtained the raw fastq files corresponding to each of the 4 studies from the SRA archive (see Supplementary table 1 for accession numbers). We identified the specific adapter sequences and barcodes from each dataset and trimmed them together with low-quality tails using Flexbar [^1^](https://paperpile.com/c/eT0xz5/rW5DM). The reads were further collapsed using fastx-toolkit[^2^](https://paperpile.com/c/eT0xz5/S8LLG) and mature human miRNAs annotated in miRBAse version 22[^3^](https://paperpile.com/c/eT0xz5/aZ46Q) were quantified using the quantifier.pl module from the miRDeep2 software, version 2.0.1.0[^4^](https://paperpile.com/c/eT0xz5/Di2tU), default options. The quantifier.pl module maps miRNAs to a reference set of miRNAs obtained by aligning the mature miRNAs from miRBase against their corresponding hairpins, and using the mature miRNAs plus their flanking +- 3 nucleotides from the alignment as a reference set for mapping the RNA-Seq reads. Differential expression (DE) analysis was performed using the DESeq2 package version 1.22.2 [^5^](https://paperpile.com/c/eT0xz5/AA99g), using mature miRNA counts as input. A simple control-treatment design was used, with the Wald test for log2FC different from 0 and the default Benjamini-Hochberg FDR control method.

For the microarray datasets, we used the GeoQuery package (v.2.50.5) [^6^](https://paperpile.com/c/eT0xz5/ZmfKE) to retrieve the already normalized intensities, and the Limma package (v.3.38.3) [^7^](https://paperpile.com/c/eT0xz5/lNkl2) for differential expression analysis, with a simple model design based on condition (UC, CD, or Control). Both microarray datasets included in our analysis were based on the Affymetrix Multispecies miRNA-2 Array platform (GPL14613). In order to synchronize the miRNA identifiers from the chip annotation to the current miRBase v.22 identifiers, we mapped the mature miRNA sequences provided in the microarray annotation to the set of mature human miRNAs in miRBase and retained only unique sequences for further analysis. Probe sequences provided for the Affymetrix Multispecies miRNA-2 Array platform (GPL14613) were aligned using blast (seed=16, no mismatches) to the set of human mature miRNAs from MiRBase release 22. When the same probe mapped to different miRNAs, only the longest hit was retained. When a miRNA matched multiple probes, a pre-filtering of hits was performed based on the blast e-value/length. In order to avoid biases in the multiple testing correction, only the probe with the highest average expression was retained for further analysis, when multiple probes matched one miRNA.

For some of the datasets, only specific subsets of samples were selected, depending on disease stratification (see Supplementary table 1). The average mapping rates to the reference set of miRBase mature miRNAs, reported by miRDeep2, ware 0.70% for dataset GSE89667, 0.51% for GSE114591 and 0.73% for GSE84779. For dataset GSE66208, the mapping rates were highly variable across the different samples, ranging between 0.06 to 0.54%, with an average of 0.27%. We removed from further analysis the samples with fewer than 1M mapped reads (4 samples).

## Meta-analysis

For the 3 UC datasets and 4 CD datasets (supplementary data, Sheet 8), we combined p-values using the logit method, and then applied the Benjamini Hochberg correction. Full meta-analysis results can be found in the supplementary data, Sheet 9 and 10. The sets of DE miRNAs in UC and CD were obtained by requiring that miRNAs are DE with a pvalue < 0.05 in at least 2 datasets and a combined adjusted pvalue < 0.05; miRNAs DE in opposite directions across the different datasets were excluded. For each dataset, we assigned each miRNA with an expression rank, by sorting the full set of miRNAs in the reverse order of their average expression across samples. For each miRNA, we averaged the expression ranks across the 3 UC datasets or the 4 CD datasets, to obtain the average expression rank. Similarly, we obtained the average log fold change in UC and CD. We used these values to filter for top DE miRNAs, as well as for our Cytoscape visualization.

## References:

1. [Dodt, M., Roehr, J. T., Ahmed, R. & Dieterich, C. FLEXBAR-Flexible Barcode and Adapter Processing for Next-Generation Sequencing Platforms. *Biology*  **1**, 895–905 (2012).](http://paperpile.com/b/eT0xz5/rW5DM)

2. [Gordon, A., Hannon, G. J. & Others. Fastx-toolkit. *FASTQ/A short-reads preprocessing tools (unpublished) http://hannonlab. cshl. edu/fastx_toolkit* **5**, (2010).](http://paperpile.com/b/eT0xz5/S8LLG)

3. [Kozomara, A. & Birgaoanu, M. miRBase: from microRNA sequences to function. *Nucleic acids* (2018).](http://paperpile.com/b/eT0xz5/aZ46Q)

4. [Friedländer, M. R., Mackowiak, S. D., Li, N., Chen, W. & Rajewsky, N. miRDeep2 accurately identifies known and hundreds of novel microRNA genes in seven animal clades. *Nucleic Acids Res.* **40**, 37–52 (2012).](http://paperpile.com/b/eT0xz5/Di2tU)

5. [Love, M. I., Huber, W. & Anders, S. Moderated estimation of fold change and dispersion for RNA-seq data with DESeq2. *Genome Biol.* **15**, 550 (2014).](http://paperpile.com/b/eT0xz5/AA99g)

6. [Davis, S. & Meltzer, P. S. GEOquery: a bridge between the Gene Expression Omnibus (GEO) and BioConductor. *Bioinformatics* **23**, 1846–1847 (2007).](http://paperpile.com/b/eT0xz5/ZmfKE)

7. [Ritchie, M. E. *et al.* limma powers differential expression analyses for RNA-sequencing and microarray studies. *Nucleic Acids Res.* **43**, e47 (2015).](http://paperpile.com/b/eT0xz5/lNkl2)
